# ﻿Two new species of *Ganoderma* (Ganodermataceae, Basidiomycota) from Southwest China

**DOI:** 10.3897/mycokeys.106.121526

**Published:** 2024-06-19

**Authors:** Jun He, Xiao-Jun Li, Wan-Zhong Tan, Xiao-Qu Wu, Dan Wu, Zong-Long Luo, Qi Wu Zhou, E-Xian Li, Shu-Hong Li

**Affiliations:** 1 College of Biotechnology and Engineering, West Yunnan University, Lincang 677000, Yunan, China West Yunnan University Lincang China; 2 Biotechnology and Germplasm Resources Institute, Yunnan Academy of Agricultural Sciences, Kunming 650205, Yunnan, China Biotechnology and Germplasm Resources Institute, Yunnan Academy of Agricultural Sciences Kunming China; 3 School of Agriculture, Yunan University, Kunming 650504, Yunan, China Yunan University Kunming China; 4 College of Agriculture and Biological Science, Dali University, Dali 671003, Yunnan, China Dali University Dali China

**Keywords:** 2 new taxa, Ganodermataceae, Morphology, Phylogeny, Taxonomy

## Abstract

*Ganoderma* is a large and diverse genus containing fungi that cause white rot to infect a number of plant families. This study describes *G.phyllanthicola* and *G.suae* as new species from Southwest China, based on morphological and molecular evidence. *Ganodermaphyllanthicola* is characterized by dark brown to purplish black pileus surface with dense concentric furrows, pale yellow margin, irregular pileipellis cells, small pores (5–7 per mm) and ellipsoid to sub-globose basidiospores (8.5–10.0 × 6.0–7.5 µm). *Ganodermasuae* is characterized by reddish brown to oxblood red pileus surface and lead gray to greyish-white pore surface, heterogeneous context, wavy margin and almond-shaped to narrow ellipsoid basidiospores (8.0–10.5 × 5.0–7.0 μm). The phylogeny of *Ganoderma* is reconstructed with multi-gene sequences: the internal transcribed spacer region (ITS), the large subunit (nrLSU), translation elongation factor 1-α gene (TEF-1α) and the second subunit of RNA polymerase II (RPB2). The results show that *G.suae* and *G.phyllanthicola* formed two distinct line-ages within *Ganoderma*. Descriptions, illustrations and phylogenetic analyses results of the two new species are presented.

## ﻿Introduction

Ganodermataceae is one of the main families of polypores with fourteen accepted genera: *Amauroderma* Murrill, *Amaurodermellus* Costa-Rezende, Drechsler-Santos & Góes-Neto, *Cristataspora* Robledo & Costa-Rezende, *Foraminispora* Robledo, Costa-Rez. & Drechsler-Santos, *Furtadoella* B.K. Cui & Y.F. Sun, *Ganoderma* P. Karst., *Haddowia* Steyaert, *Humphreya* Steyaert, *Magoderna* Steyaert, *Neoganoderma* B.K. Cui & Y.F. Sun, *Sanguinoderma* Y.F. Sun, D.H. Costa & B.K. Cui, *Sinoganoderma* B.K. Cui, J.H. Xing & Y.F. Sun, *Tomophagus* Murrill and *Trachydermella* B.K. Cui & Y.F. Sun (Costa-Rezende et al. 2020; Sun et al. 2022), of which most species are classified in the genus *Ganoderma*.

The word *Ganoderma* is derived from the Greek words “Gano”, meaning “shiny”, and “derma”, meaning “skin” (Loyd et al. 2018). The genus *Ganoderma* (Polyporales, Basidiomycota) was described by Fries (1821) based on *Polyporuslucidus* (Curtis) Fr. and typified by *Ganodermalucidum* (Curtis) P. Karst. from Europe (Fries 1821; Karsten 1881). *Ganoderma* is a globally distributed genus of wood-decaying fungi that encompass important species for forestry, medicine, food, and cultural traditions, in which morphological delimitation has been challenging due to its large plasticity and wide distribution across various regions (Luangharn et al. 2021). In the past few decades, DNA or amino acid sequence analyses have provided effective tools for taxonomists to combine data. These modern techniques have helped to clarify the distribution of different species complexes in the genus *Ganoderma*, and have revealed some instances of misidentification (Kinge et al. 2015; Fryssouli et al. 2020).

The genus is characterized by laccate or non-laccate basidiocarps, sessile to stipitate basidiomata, white to pale yellow margin, and red-brown colored truncate double-walled basidiospores, an apical germinal pore, thin and colourless external wall (exosporium), with a brown to dark brown interwall pillars (endosporium), and the ability to cause white rot in woody plants (Karsten 1881; Moncalvo and Ryvarden 1997). Furthermore, these species hold different characteristics, such as the shape and the color of the fruit body, host specificity, and geographical origin, which are used to identify individual members of the species. The species concept in the genus *Ganoderma* is thus not universally accepted nor well established due to the highly variable morphological features of the species (Wang et al. 2014; Náplavová et al. 2020).

Currently, based on credible morphological and phylogenetic evidence, 191 species of *Ganoderma* have been described worldwide (He et al. 2022; Sun et al. 2022; Vinjusha et al. 2022; Cabarroi-Hernández et al. 2023). *Ganoderma* is economically important, due to the fact that members of the genus are regarded as valuable medicinal mushrooms (Hapuarachchi et al. 2018a). Several *Ganoderma* species are known to be prolific sources of a high number of natural bioactive compounds such as polysaccharides, triterpenoids, sterols, and secondary metabolites (Richter et al. 2015). Approximately 45 species of *Ganoderma* are recorded in Chinese Fungi (Sun et al. 2022), of which *Ganodermalucidum* “lingzhi” and *G.sinense* which used to be listed in Chinese Pharmacopeia to prevent and treat many diseases and are listed in Chinese Pharmacopeia, and which are included in the homologous list of medicine and food (Li et al. 2018). Furthermore, *Ganoderma* was included in the American Herbal Pharmacopoeia and Therapeutic Compendium (Hapuarachchi et al. 2018b). They are commonly named as “Lingzhi” or “Rui–zhi” in China, “Youngzhi” in Korea, “Reishi” in Japan and “Ganoderma” in the USA (Liu et al. 2015). These natural bioactive compounds are used to treat and remedy many pathological diseases, including traditional medicine for treating neurasthenia, debility of prolonged illness, insomnia, arthritis, asthma, anorexia, dizziness, chronic hepatitis, hypercholesterolemia, mushroom poisoning, coronary heart disease, hypertension, prevention of acute mountain sickness, deficiency fatigue’, carcinoma, and bronchial cough in the elderly (Wang et al. 2020). In addition, *Ganoderma* products come in the form of various commercial products of *Ganoderma* such as powders, dietary supplements, coffee, tea, spore products, drinks, syrup, toothpaste, soap, lotion, and capsules, and have been commercialized as effective food and drug supplements for health benefits (Lai et al. 2004).

Southwest China contains some of the highest concentrations of fungal biodiversity in the world, and Yunnan Province, in particular, has a varied topography, environmental conditions, and a variety of habitats for a diverse range of fungi (He et al. 2022). Despite the advancement in taxonomic studies of *Ganoderma* species diversity, many novel species are still being discovered (He et al. 2021; He et al. 2022). During our investigations of macrofungi in Southwest China, a couple of specimens of *Ganoderma* were collected. In the current study, the phylogenetic analyses of *Ganoderma* were carried out based on the combined sequence dataset of ITS + nLSU + TEF1–α + RPB2 gene regions. Subsequent morphological and molecular studies uncovered two undescribed species. These species are illustrated and described below.

## ﻿Materials and methods

### ﻿Specimen collection

During the rainy season from June 2019 to September 2023, four *Ganoderma* specimens were collected in southwest China. They were photographed in the field, then macro-mophology was described on fresh basidiomata, on the same day of collection. Specimens were there after thoroughly dried at 45 °C (Hu et al. 2022), in a thermostatic drier, stored in sealed plastic bags, and deposited in the herbarium of Kunming Institute of Botany, Chinese Academy of Sciences Academia Sinica (KUN-HKAS).

### ﻿Morphological studies

Colour codes were determined following Kornerup and Wanscher (1978). For microscopic characteristics, anatomical and cytological characteristics including basidia, basidiospores, hyphal system, and pileipellis were observed and photographed using a Nikon ECLIPSE Ni-U microscope (Nikon, Japan) at magnifications up to × 1000. Tarosoft(R) Image Frame Work (IFW) was used for the measurement of photomicrographs, and Adobe Photoshop CS5 software was used to process images for making photo plates (He et al. 2021).

The following abbreviations are used: IKI = Melzer’s reagent, IKI– = neither amyloid nor dextrinoid, KOH = 10% potassium hydroxide, CB = Cotton Blue, CB+ = cyanophilous. The notation [n/m/p] specifies that measurements were made on “n” basidiospores from “m” basidiomata and “p” collections. Basidiospore dimensions are given as (a) b–*av*– c (d). Where a and d refer to the lower and upper extremes of all measurements, respectively, b-c the range of 95% of the measured values, L = mean spore length (arithmetic average of all spores), W = mean spore width (arithmetic average of all spores), *Q* is the length/width ratio of basidiospores, *Qm* denotes the average of n measured basidiospores and SD is their standard deviation. Results are presented as *Q* = *Qm* ± SD.

### ﻿DNA extraction, PCR amplification, and sequencing

Genomic DNA was extracted from dry specimens using the Ezup Column Fungi Genomic DNA Purification Kit following manufacturer instructions. Primers pairs for PCR were respectively ITS1F/ITS5 (White et al. 1990), LR5/LR0R (Liu et al. 1999), TEF1–983 / TEF1–1567R (Matheny et al. 2007), and RPB2–6f / fRPB2–7cR (Liu et al. 1999), respectively. Primer sequences are available in the WASABI database at the AFTOL website (aftol.org). The PCR mixture was prepared in a 30 μL final volume, with 15 μL 2× Taq Plus Master Mix II (Sangon Biotechnology Co., Kunming, China), 12 μL ddH2O, 0.5 μL 10 μM of forward and reverse primers, 2 μL DNA. The PCR thermal cycle program for ITS and nrLSU amplification was conducted using the following profiles: 94 °C for 5 min, 35 cycles of 94 °C for 30 s, 53 °C for 50 s, 72 °C for 1 min, and 72 °C for 10 min. The PCR cycling for TEF1-α was as follows: initial denaturation at 94 °C for 5 min, followed by 35 cycles at 94 °C for 30 s, 55 °C for 30 sec and 72 °C for 1 min, and 72 °C for 10 min. The PCR cycling for RPB2 was as follows: initial denaturation at 94 °C for 5 min, followed by 35 cycles at 94 °C for 30 s, 50 °C for 50 s and 72 °C for 1 min, and 72 °C for 10 min. PCR products were checked on 1% agarose gels stained with ethidium bromide under UV light. The PCR products were purified and sequenced by the Sangon Biotech Limited Company (Shanghai, China). Raw DNA sequences were assembled and edited in Sequencher 4.1.4, and the assembled DNA sequences were deposited in GenBank (Table 1).

**Table 1.** Names, voucher numbers, origins, and their corresponding GenBank accession numbers of the taxa used in the phylogenetic analyses. The new species sequences generated sequences is show in bold, after the species name and the type specimens show “T” after the number.

Species	Voucher/strain	Origin	GenBank accession numbers			
			ITS	nLSU	TEF1–α	RPB2
*Ganodermaacaciicola*	Cui16815^T^	Australia	MZ354895	MZ355005	–	MZ245384
*G_acaciicola*	Cui16813	Australia	MZ354893	MZ355003	–	MZ245382
*G.artocarpicola*	HL173^T^	Yunnan, China	ON994239	OP456495	OP508442	OP508428
*G.artocarpicola*	HL188	Yunnan, China	ON994240	OP380253	OP508441	OP508427
*G.aridicola*	Dai12588^T^	South Africa	KU572491	–	KU572502	–
*G.austroafricanum*	CBS138724^T^	South Africa	KM507324	KM507325	–	MK611970
*G.austroafricanum*	CMW25884	South Africa	MH571693	–	MH567296	–
*G.boninense*	WD2085	Japan	KJ143906	–	KJ143925	KJ143965
*G.boninense*	WD2028	Japan	KJ143905	KU220015	KJ143924	KJ143964
*G.bubalinomarginatum*	Dai20075 ^T^	Guangxi, China	MZ354926	MZ355010	MZ221637	MZ245388
*G.bubalinomarginatum*	Dai20074	Guangxi, China	MZ354927	MZ355040	MZ221638	MZ245389
*G.carocalcareum*	DMC513	Cameroon	EU089970	–	–	–
*G.carocalcareum*	DMC322 ^T^	Cameroon	EU089969	–	–	–
*G.casuarinicola*	HKAS104639	Thailand	MK817650	MK817654	MK871328	MK840868
*G.casuarinicola*	Dai16336 ^T^	Guangdong, China	MG279173	–	MG367565	MG367508
*G.concinnum*	Robledo3235	Brazil	MN077523	MN077557	–	–
*G.concinnum*	Robledo3192	Brazil	MN077522	MN077556	–	–
*G.curtisii*	CBS100132	NC, USA	JQ781849	–	KJ143927	KJ143967
*G.curtisii*	CBS100131	NC, USA	JQ781848	–	KJ143926	KJ143966
*G.destructans*	CBS139793 ^T^	South Africa	NR132919	NG058157	–	–
*G.destructans*	Dai16431	South Africa	MG279177	–	MG367569	MG367512
*G.dunense*	CMW42150	South Africa	MG020249	–	MG020228	–
*G.dunense*	CMW42157 ^T^	South Africa	MG020255	–	MG020227	–
*G.ecuadorense*	URM89449	Ecuador	MK119828	MK119908	MK121577	MK121535
*G.ecuadorense*	URM89441	Ecuador	MK119827	MK119907	MK121576	MK121534
*G.enigmaticum*	Dai15971	Africa	KU572487	–	KU572497	MG367514
*G.enigmaticum*	Dai15970	Africa	KU572486	–	KU572496	MG367513
*G.heohnelianum*	Cui13982	Guangxi, China	MG279178	–	MG367570	MG367515
*G.heohnelianum*	Dai11995	Yunnan, China	KU219988	KU220016	MG367550	MG367497
*G.hochiminhense*	MFLU19_2225	Vietnam	MN396662	MN396391	MN423177	–
*G.hochiminhense*	MFLU19_2224 ^T^	Vietnam	MN398324	MN396390	MN423176	–
*G.lingzhi*	Dai20895	Liaoning, China	MZ354904	MZ355006	MZ221668	MZ245413
*G.lingzhi*	HL56	Yunnan, China	ON994247	OP380262	–	OP508423
*G.martinicense*	246TX	TX, USA	MG654185	–	MG754737	MG754858
*G.martinicense*	LIPSWMart0855 ^T^	Martinique, France	KF963256	–	–	–
***G.suae***	**L4651** ^T^	**Yunnan, China**	**PP869243**	**PP869250**	**PP894782**	**PP894784**
***G.suae***	**L4817**	**Yunnan, China**	**PP869244**	**PP869251**	**PP894783**	–
*G.mexicanum*	MUCL55832	Martinique	MK531815	–	MK531829	MK531839
*G.mexicanum*	MUCL49453	Martinique	MK531811	–	MK531825	MK531836
*G.mirabile*	Cui18271	Malaysia	MZ354958	MZ355067	MZ221672	MZ345729
*G.mirabile*	Cui18283	Malaysia	MZ354959	MZ355069	MZ221673	MZ345730
*G.mizoramense*	UMNMZ5	India	KY643751	KY747490	–	–
*G.mizoramense*	UMNMZ4T	India	KY643750	–	–	–
*G.multipileum*	Cui13597	Hainan, China	MZ354899	MZ355043	MZ221675	MZ345732
*G.multipileum*	L4989	Yunnan, China	ON994249	OP380264	OP508447	OP508432
*G.multiplicatum*	CC8	China	KU569515	KU570915	–	–
*G.multiplicatum*	Dai17395	Brazil	MZ354903	–	MZ221678	MZ345734
*G.multiplicatum*	SPC9	Brazil	KU569553	KU570951	–	–
*G.multiplicatum*	URM83346	Brazil	JX310823	JX310837	–	–
*G.myanmarense*	MFLU19_2167 ^T^	Myanmar	MN396330	MN428672	–	–
*G.myanmarense*	MFLU19_2169	Myanmar	MN396329	MN398325	–	–
*G.nasalanense*	GACP17060211 ^T^	Laos	MK345441	MK346831	–	–
*G.nasalanense*	GACP17060212	Laos	MK345442	MK346832	–	–
*G.orbiforme*	HL43	Yunnan, China	ON994250	OP380265	OP508435	–
*G.orbiforme*	TNM F0018838	China	JX840350	–	–	–
*G.parvulum*	MUCL52655	Guiana, French	MK554770	–	MK554717	MK554755
*G.parvulum*	MUCL47096	Cuba	MK554783	–	MK554721	MK554742
*G.philippii*	Cui14443	Hainan, China	MG279188	–	MG367578	MG367524
*G.philippii*	MFLU19/2222	Thailand	MN401410	MN398326	MN423174	–
*G.polychromum*	330OR	OR, USA	MG654196	–	MG754742	–
*G.polychromum*	MS343OR	OR, USA	MG654197	–	MG754743	–
*G.ravenelii*	MS187FL	FL, USA	MG654211	–	MG754745	MG754865
*G.ravenelii*	NC_8349	USA	AY456341	–	–	–
*G.resinaceum*	LGAM462	Greece	MG706250	MG706196	MG837858	MG837821
*G.resinaceum*	LGAM448	Greece	MG706249	MG706195	MG837857	MG837820
*G.resinaceum*	MUCL38956	Netherlands	MK554772	–	MK554723	MK554747
*G.resinaceum*	MUCL52253	France	MK554786	–	MK554737	MK554764
*G.rodriguezii*	M–11926	Cuba	OQ079179	–	–	–
*G.rodriguezii*	269TX	USA	MG654352	–	–	–
*G.ryvardenii*	HKAS58053 ^T^	South Africa	HM138670	–	–	–
*G.ryvardenii*	HKAS58054	South Africa	HM138671	–	–	–
*G.sessile*	113FL	FL, USA	MG654307	–	MG754748	MG754867
*G.sessile*	111TX	TX, USA	MG654306	–	MG754747	MG754866
*G.sichuanense*	Cui16343	China	MZ354928	MZ355011	MZ221692	MZ345741
*G.sichuanense*	Dai19651	Sri Lanka	MZ354929	MZ355031	MZ221693	MZ345742
*G.sinense*	Wei5327	Hainan, China	KF494998	KF495008	KF494976	MG367529
*G.sinense*	HL109	Yunnan, China	ON994252	OP380267	OP508438	OP508425
*G.steyaertanum*	MEL2382783	Australia	KP012964	–	–	–
*G.steyaertanum*	6WN 20B	Indonesia	KJ654462	–	–	–
***G.phyllanthicola***	**L4948 ^T^**	**Yunnan, China**	**PP869245**	**PP869252**	–	–
***G.phyllanthicola***	**HL308**	**Yunnan, China**	**PP869246**	**PP869253**	–	–
*G.thailandicum*	HKAS104640 ^T^	Thailand	MK848681	MK849879	MK875829	MK875831
*G.thailandicum*	HKAS104641	Thailand	MK848682	MK849880	MK875830	MK875832
*G.tropicum*	Dai16434	Hainan, China	MG279194	MZ355026	MG367585	MG367532
*G.tropicum*	HL186	Yunna, China	ON994253	OP380268	OP508440	–
*G.tuberculosum*	GVL40	Veracruz, Mexico	MT232634	–	–	–
*G.tuberculosum*	JV1607_62	Costa Rica	MZ354944	MZ355087	MZ221710	–
*G.weberianum*	CBS21936	Philippines	MK603804	–	MK611974	MK611972
*G.weberianum*	Dai19673	China	MZ354930	MZ355032	MZ221712	MZ358829
*G.wiiroense*	UMN21GHA ^T^	Ghana	KT952363	KT952364	–	–
*G.wiiroense*	UMN20GHA	Ghana	KT952361	KT952362	–	–
*G.zonatum*	FL03	FL_USA	KJ143922	–	KJ143942	KJ143980
*G.zonatum*	FL02	FL_USA	KJ143921	–	KJ143941	KJ143979
*Amaurodermarugosum*	Cui9011	Guangdong, China	KJ531664	–	KU572504	MG367506

### ﻿Sequencing and sequence alignment

Sequences newly generated in this study and sequences obtained from GenBank (Table 1) were analyzed. The related sequences were determined by using a BLAST search to reveal the closest matches with taxa in *Ganoderma* and recent relevant publications (Sun et al. 2022). Sequences were aligned using MAFFT v.7 (http://mafft.cbrc.jp/alignment/server/) (Katoh and Standley 2013) and then checked visually and manually optimized using BioEdit v.7.0.9 (Hall 1999), to allow maximum alignment and minimize gaps. Ambiguous regions were excluded from the analyses and gaps were treated as missing data. The phylogeny website tool “ALTER” (Glez-Peña et al. 2010) was used to convert the alignment fasta file to Phylip format for RAxML analysis and AliView and PAUP 4.0 b 10 were used to convert the alignment fasta file to a Nexus file for Bayesian analysis (Swofford 2003).

### ﻿Phylogenetic analyses

A maximum likelihood (ML) analysis was performed at the CIPRES web portal (Miller et al. 2010) using RAxML v.8.2.12 as part of the “RAxML-HPC2 on TG” tool (Miller et al. 2010). A general time-reversible model (GTR) was applied with a discrete gamma distribution and four rate classes. Fifty thorough ML tree searches were conducted out in RAxML v.8.2.11 under the same model. One thousand non-parametric bootstrap iterations were run with the GTR model and a discrete gamma distribution. The resulting replicates were plotted onto the best scoring tree obtained previously. Since no supported conflict (BS ≥ 60%) was detected among the topologies, the four single-gene alignments were concatenated using SequenceMatrix (Vaidya et al. 2011).

The Bayesian analyses were performed using PAUP v.4.0b10 and MrBayes v.3.2 (Ronquist et al. 2012), and the best-fit model of sequences evolution was estimated via MrModeltest 2.3 (Guindon and Gascuel 2003; Nylander 2004; Darriba et al. 2012). Markov Chain Monte Carlo (MCMC) sampling approach was used to calculate posterior probabilities (PP) (Rannala and Yang 1996). Bayesian analyses of six simultaneous Markov chains were run for one million generations and trees were sampled every 100^th^ generation with a total of 10,000 trees. The first 2000 trees were discarded and the remaining trees were used for calculating posterior probabilities in the majority rule consensus tree.

Phylogenetic trees were visualized using FigTree v1.4.4 (http://tree.bio.ed.ac.uk/software/figtree/), and editing and typesetting was done using Adobe Illustrator CS5 (Adobe Systems Inc., USA). Sequences derived in this study were deposited in GenBank (http://www.ncbi.nlm.nih.gov). The final sequence alignments and the phylogenetic trees are available at TreeBase (http://www.treebase.org, accession number: 31439).

## ﻿Results

### ﻿Phylogenetic analyses

**Figure 1.**
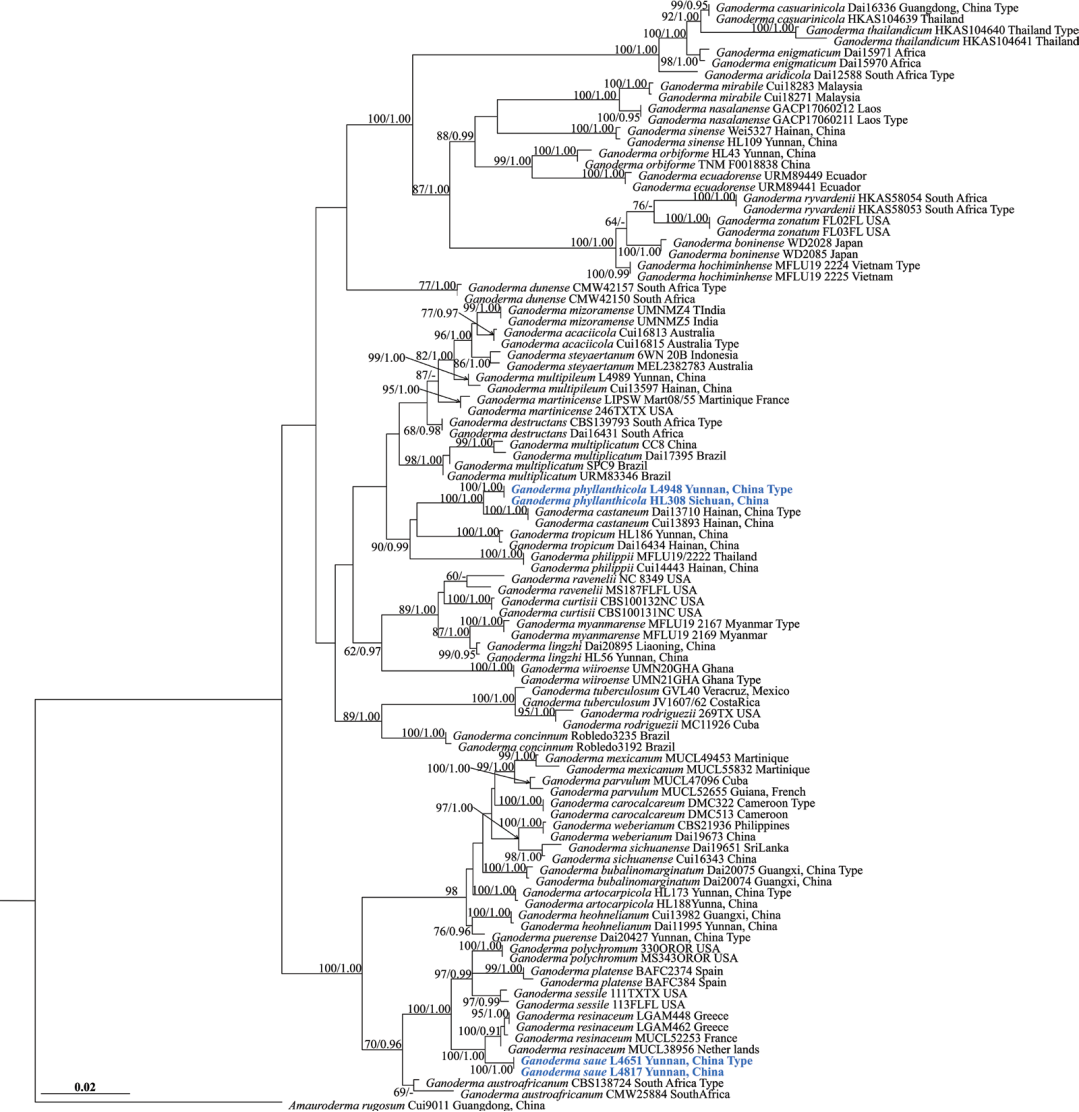
Maximum likelihood (ML) tree based on combined ITS + nrLSU + TEF1-α + RPB2 sequence data. Bootstrap support values with a maximum likelihood (ML) equal to or greater than 60% and Bayesian posterior probabilities (PP) equal to or greater than 0.90 are given above the nodes, shown as “ML/PP”. New species are indicated in bold blue.

In this study, eleven sequences were newly generated from specimens of *Ganoderma* spp. and deposited in GenBank (Table 1), all collected from Yunnan Province, China. The dataset comprised combined ITS + nrLSU + TEF1-α + RPB2 sequences data from 94 specimens, representing 46 taxa in Ganodermataceae. The aligned dataset comprised 2633 characters including gaps (ITS: 1–576; nrLSU: 577–1423; TEF1-α: 1424–1959; RPB2: 1969–2633) of which *Amaurodermarugosum* Cui 9011 as the outgroup taxon (Fig. 1, Sun et al. 2020). The likelihood of the final tree was evaluated and optimized under GAMMA. The best RAxML tree with a final likelihood value of -13209.788540 is presented (Fig. 1). The matrix had 855 distinct alignment patterns, with 37.25% undetermined characters or gaps. Estimated base frequencies were as follows: A = 0.223857, C = 0.250309, G = 0.275931, T = 0.249903; substitution rates AC = 1.104623, AG = 5.821128, AT = 1.180576, CG = 1.273064, CT = 8.821984, GT = 1.000000, α = 0.169579, Tree-Length: 1.075203. Best model for the ITS + nLSU + TEF1-α + RPB2 dataset estimated and applied in the Bayesian analysis were HKY+I+G for ITS and RPB2 (Lset nst=2, rates=invgamma; Prset statefreqpr=dirichlet (1,1,1,1)), GTR+I+G for nrLSU and TEF1-α (Lset nst=6, rates=invgamma; Prset statefreqpr=dirichlet (1,1,1,1). ML analysis resulted in a similar and applied in the Bayesian in equal frequency of nucleotides. Bootstrap support values with a maximum likelihood (ML) equal to or greater than 60%, and Bayesian posterior probabilities (PP) equal to or greater than 0.90 are given above the nodes (Fig. 1).

The phylogeny demonstrated that our four *Ganoderma*-like specimens were clustered into two different lineages with high support, represented two new species, *G.phyllanthicola* (100% BS and 1.00 BP; Fig. 1) and *G.suae* (100% BS and 1.00 BPP; Fig. 1). *Ganodermaphyllanthicola* sp. nov. clustered as a sister clade with *G.castaneum* BK Cui, JH Xing & YF Sun *G.tropicum* (Jungh.) Bres. and *G.philippii* Bres. & Henn. ex Sacc. with strong statistical support (90%ML/0.99PP, Fig. 1), but forming a distinct lineage. *Ganodermasuae* sp. nov. was sister to *G.resinaceum* Boud. with high statistical support (100%ML/1.00PP, Fig. 1).

### ﻿Taxonomy

#### ﻿ Ganoderma phyllanthicola

Taxon classificationFungiPolyporalesPolyporaceae

J. He & S.H. Li sp. nov.

B97E4210-9A58-5ACD-AF98-46B151900A39

853508

Fig. 2

##### Diagnosis.

Differs from other species in the genus by its sessile and coriaceous basidiomata, dark brown to purplish black pileus surface with dense concentric furrows, pale yellow margin, irregular pileipellis cells, broadly ellipsoid to subglobose basidiospores and truncated apex, exospore walls smooth, endospore walls with dense spinules.

##### Etymology.

The epithet ‘*phyllanthicola*’ refers to the host tree genus *Phyllanthus*.

##### Holotype.

China. Yunnan Province., Honghe City, Mengzi County, on living tree of *Phyllanthusemblica*, alt. 1685 m, Jun He, 26 August. 2019, L4948(HKAS 123776).

##### Description.

***Basidiomata*** annual, sessile and broadly attached, coriaceous, hard corky to woody hard. ***Pileus*** single or dimidiate, sub-circular, flabelliform to shell-shaped, applanate, projecting up to 22 cm, 12 cm wide and 1.9 cm thick at base. Pileus surface dark brown(8F8), purplish black(8F3) to reddish brown(6F8) and covered by a thin hard crust, laccate, glabrous and shiny, with dense concentric furrows. ***Margin*** pale yellow(4A3) to generally concolorous, entire, subacute, slightly wavy. ***Context*** up to 0.8 cm thick, homogeneous, cinnamon brown(6D7) to chestnut brown(8E5), with black melanoid lines, hard corky. ***Tubes*** 0.5–1.1 cm long, concolorous with the base of the context, corky, unstratified. ***Pores*** 5–7 per mm, circular to subcircular, dissepiments slightly thick, entire; pores surface greyish white(2B1) when fresh, orange grey(5B2) to pale brown(6D6) when bruising and drying.

**Figure 2.**
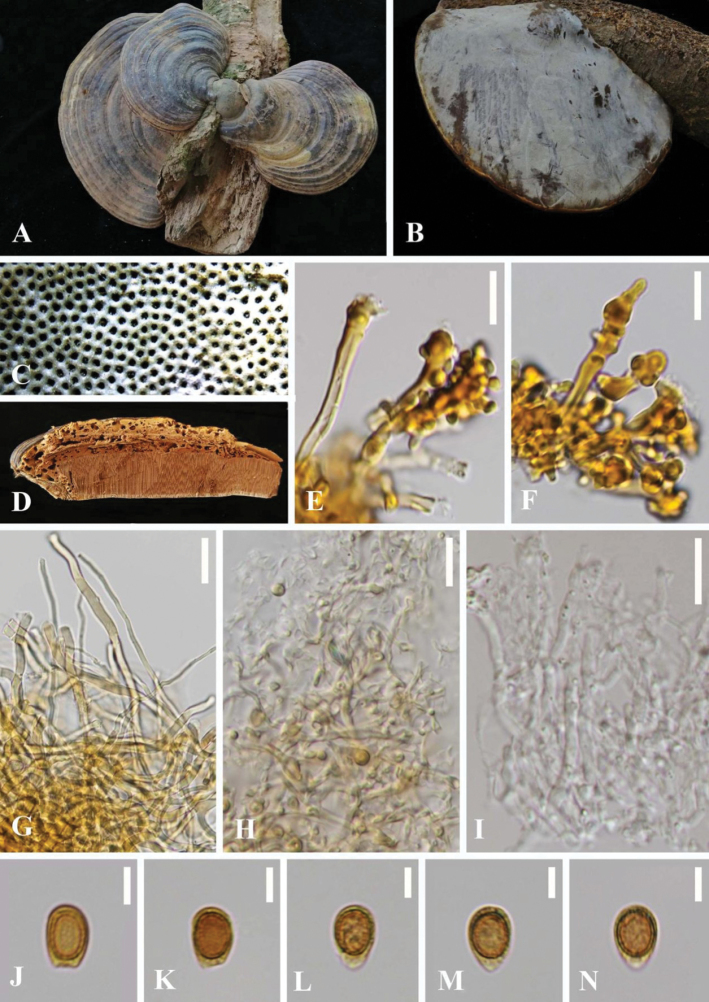
*Ganodermaphyllanthicola* (HKAS 123776, holotype) **A, B** basidiomata **C** pore surface **D** cut side of pileus **E, F** sections of pellis **G** skeletal hyphae from context **H** binding hyphae from context **I** generative hyphae from tubes **J–N** basidiospores. Scale bars: 20 μm (**G**); 10 μm (**E, F, H, I**); 5 μm (**J–N**).

***Hyphal system trimitic*.** Generative hyphae 1.0–2.0 μm in diameter, colorless, thin-walled, with clamps connections; skeletal hyphae 2.0–5.0 μm in diameter, thick-walled with a wide to narrow lumen or sub-solid, arboriform with few branches, yellowish to golden yellow; binding hyphae 1.0–3.0 μm in diameter, thick-walled, branched and flexuous, pale yellow, scarce; all the hyphae IKI–, CB+; tissues darkening in KOH.

***Pileipellis*** a crustohymeniderm, cells 15–33 × 5–9 μm, thick-walled to sub-solid, composed of irregular, narrowly clavate end cells, straight to flexuose, smooth or with a few small apical protuberances, yellowish to golden-yellow, sometimes with apical granulations, apex slightly amyloid.

***Basidiospores*** ellipsoid to subglobose, apex truncated or subacute, yellowish to yellowish brown, IKI–, CB+, inamyloid; double-walled with distinctly thick walls, exospore wall smooth, endospore walls with interwall pillars; (40/2/2) (8.5) 9.0–9.6–10.0 (11.0) × 6.0–6.8–7.0 (7.5) µm, L = 9.65 µm, W = 6.75 µm, *Q* = (1.24) 1.38–1.52 (1.55), *Q*m = 1.43 ± 0.07 (including myxosporium). ***Basidia*** not observed.

##### Additional specimens examined.

China, Sichuan Province, Panzhihua City, Miyi County, on a decaying tree of *Phyllanthus* sp., alt. 1035 m, Jun-He, 15 August 2023, HL308.

##### Notes.

In the phylogenetic analyses, *Ganodermaphyllanthicola* is clustered as a sister taxon to *G.castaneum* with strong statistical support (100% ML and 1.00PP, Fig. 1). Morphologically, both species share similar characteristics of the sessile basidiomata and non-stratified tubes. However, *G.castaneum* differs from *G.phyllanthicola* in having buff and obtuse pileus margin, regular palisade pileipellis, heterogeneous context, smaller basidiospores (6.2–8.5 × 4.2–6.3 μm) with smooth endospore walls, Sun et al. 2022). *Ganodermatropicum* and *G.philippii* have similar homogeneous context, but *G.tropicum* has a stipitate basidiomata and buff pileus margin, samller basidiospores (6.8–10.0 × 4.0–6.4 μm), and (Steyaert 1972). *Ganodermaphilippii* has wavy like pileus margin and brown context with black melanoid lines, smaller and obovoid basidiospores (6.0–8.0 × 3.0–4.0 μm, Moncalvo and Ryvarden 1997).

*Ganodermaaridicola* described from South Africa is similar to *G.phyllanthicola* in the sessile basidiomata with dark brown pileus surface, homogeneous context, small pores and ellipsoid basidiospores. However, *G.aridicola* differs by the distinctly stratified tubes and lacks branched or protuberant apical cells (Xing et al. 2016). Besides, the phylogenetic analyses separated *G.aridicola* and *G.phyllanthicola* (Fig. 1). *Ganodermamultiplicatum* also has pale yellow margin and irregular pileipellis cells., but it differs from *G.phyllanthicola* by the photo brown to reddish brown pileus surface, short stipe (1.8–3 cm) and ellipsoid basidiospores (6.0–10.0 × 4.5–7.0 μm, Gottlieb and Wright 1999).

#### ﻿ Ganoderma suae

Taxon classificationFungiPolyporalesPolyporaceae

J. He & S.H. Li sp. nov.

7C41B997-89FC-50F2-81D0-549055335363

853506

Fig. 3

##### Diagnosis.

Differs from other species in the genus by its large and substipitate basidiomata, reddish brown to oxblood red pileus surface with concentric furrows and radial rugose, whitish and wavy margin, almond-shaped basidiospores, heterogeneous context and non-stratified tubes.

##### Etymology.

The epithet ‘*suae*’ refers to the Chinese mycologist Prof. Hong-Yan Su, for her great contribution to the mycology.

##### Holotype.

China. Yunnan Province., Honghe City, lvchun County, on a dead stump of a broad-leaved tree, alt. 1392 m, Jun He, 24 June 2019, L4651(HKAS 123791).

##### Description.

***Basidiomata*** annual, sessile to substipitate, and occasionally imbricate, woody-corky, light in weight. ***Pileus*** round-flabelliform to reniform, slightly convex to applanate; surface glabrous, projecting up to 15 cm, 10 cm wide and 2 cm thick at base. Pileus surface reddish brown(6F8) to oxblood red(9E7), weakly to strongly laccate, and covered by a thin hard crust, concentrically zonate or azonate. ***Margin*** whitish to generally concolorous, entire, acute to obtuse, smooth to irregularly wavy. ***Context*** up to 0.8 cm thick, heterogeneous, the upper layer greyish white(2B1), the lower layer cinnamon brown (6D7) to chestnut brown(6F7), bearing distinct concentric growth zones, without black melanoid lines, hard corky and fibrous. ***Tubes*** 0.2–1.2 cm long, grayish brown (6B3), corky, unstratified. ***Pores*** 4–6 per mm, circular to angular, dissepiments slightly thick, entire; pore surface lead gray (2D2) to greyish white (2B1) when fresh, golden grey (4C2) to soot brown(5F5) when bruising or aging. ***Stipe*** up to 4.5 cm long and 3.0 cm diam, generally short and thick, cylindrical, horizontal or lateral, fibrous to spongy, reddish brown (6F8) to dark brown (8F8), concolorous to generally darker than pileus.

**Figure 3.**
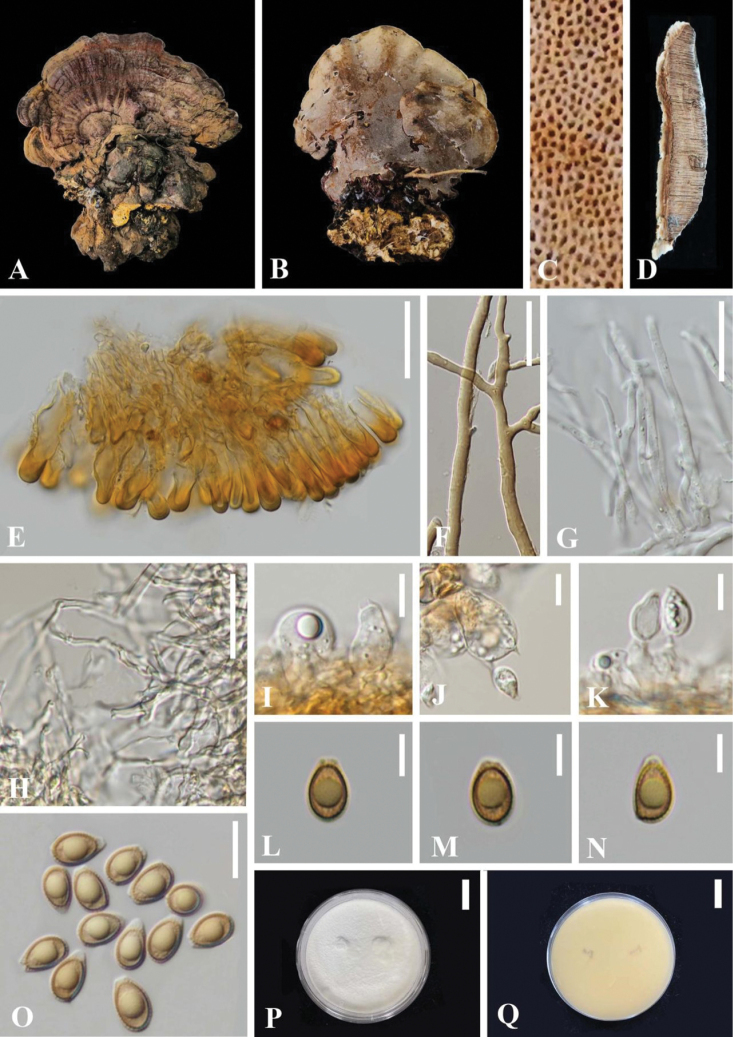
*Ganodermasuae* (HKAS 123791, holotype) **A, B** basidiomata **C** pore surface **D** cut side of pileus **E** sections of pellis **F** skeletal hyphae from context **E** generative hyphae from tubes **H** binding hyphae from context **I–K** basidia and basidioles **L–O** basidiospores. Scale bars: 30 μm (**E–H**); 10 μm (**I–K, O)**; 5 μm (**L–N**); 20 mm (**P, Q**).

***Hyphal system trimitic*.** Generative hyphae 2.0–3.0 μm in diameter, colorless, thin-walled, hyaline, unbranched, abundant, with clamp connections; skeletal hyphae 3.0–9.0 μm in diameter, thick-walled with a narrow lumen to subsolid, non-septate, moderately branched, orange yellow to golden-yellow, predominant; binding hyphae 1.0–2.0 μm in diameter, subthick-walled to solid, non-septate, frequently branched, interwoven, colourless to yellowish, scarce, notably thinner and paler than skeletal hyphae; all the hyphae IKI–, CB+; tissues darkening in KOH.

***Culture characteristics*.** Initially, white to yellowish white, pale yellow when growing, become orange white, pale orange, light orange and some reddish yellow to dark brown around the plugged circle of active mycelium after incubation for 3 weeks.

***Pileipellis*** a crustohymeniderm, cells 24–43 × 6–11 μm, thick-walled to sub-solid, apical cells narrowly clavate to clavate, slightly inflated, yellowish to golden-yellow, without granulations in the apex; negative or apex slightly amyloid.

***Basidiospores*** almond-shaped to narrow ellipsoid, apex subacute, with apical germ pore, yellowish to yellowish-brown, IKI–, CB+, inamyloid; double-walled, exospore smooth, endospore with coarse echinulate, exosporium with inter-walled pillars 0.5–0.6 μm thick; (80/4/2) (8.0) 9.0–9.7–10.5 × (5.0) 5.5–6.1–6.5 (7.0) μm, L = 9.70 µm, W = 6.10 µm, *Q* = (1.38) 1.45–1.79 (1.97), *Q*m = 1.61 ± 0.13 (including myxosporium). ***Basidia*** barrel-shaped to widely clavate, colorless, with a clamp connection and four sterigmata, thin-walled, 9–18 × 9–12 µm; basidioles pear-shaped to fusiform, colourless, thin-walled, 8–14 × 6–11 µm.

##### Additional specimens examined.

China, Yuannan Province, Lingcang City, Yun County, on a living *Quercus* sp. tree, alt. 1516 m, Jun He, 4 August 2019, L4817(HKAS 123777).

##### Notes.

Phylogenetic analyses showed that *Ganodermasuae* clusters as a sister taxon to *G.resinaceum* with good statistical support (100% ML/1.00 PP, Fig. 1). Morphologically, *G.resinaceum* differs from *G.suae* by having smaller basidiomata, reddish brown to oxblood red pileus surface and wavy margin, homogeneous context, longer pileipellis (34–59 × 6.2–9.3 μm), and larger basidiospores (11.2–12.5 × 6.5–7.4 μm, Náplavová et al. 2020; Ryvarden 2000; Torres-Torres et al. 2012).

*Ganodermazonatum* also has sessile basidiomata and a whitish pileus margin, but it differs from G. *suae* by having an apex widened to swollen of pileipellis cells (30–70 × 5–12 μm), and larger basidiospores (11.2–12.5 × 6.5–7.4 μm, Murrill 1902).

## ﻿Discussion

*Ganoderma* has long been regarded as one of the most important genera of medicinal fungi worldwide with more than 45 species described in China. To date, 36 species of *Ganoderma* have been reported from Southwest China (Yunnan, Tibet, Guizhou, and Sichuan), including 16 species originally described from China, namely *G.alpinum* B.K. Cui, J.H. Xing & Y.F. Sun, *G.artocarpicola* J. He & S.H. Li, *G.dianzhongense* J. He, H.Y. Su & S.H. Li, *G.ellipsoideum* Hapuar., T.C. Wen & K.D. Hyde 2018, *G.esculentum* J. He & S.H. Li, *G.leucocontextum* T.H. Li, W.Q. Deng, S. H. Wu, D. M. Wang & H.P. Hu, *G.mutabile* Y. Cao & H.S. Yuan, *G.obscuratum* J. He & S. H. Li, *G.ovisporum* H.D. Yang, T.C. Wen. *G.puerense* B.K. Cui, J.H. Xing & Y.F. Sun, *G.sanduense* Hapuar., T.C. Wen & K.D. Hyde, *G.sichuanense* J.D. Zhao & X.Q. Zhang, *G.subangustisporum* B.K. Cui, J.H. Xing & Y.F. Sun, *G.weixiense* Karun. & J.C. Xu, *G.yunnanense* J. He & S. H. Li and *G.yunlingense* B.K. Cui, J.H. Xing & Y.F. Sun (Zhao et al. 1983; Cao et al. 2012; Li et al. 2015; Hapuarachchi et al. 2019; He et al. 2021; He et al. 2022; Sun et al. 2022; Yang et al. 2022). During the last five years, the diversity of *Ganoderma* in Southwest China was mainly reported from Yunnan Province and Guizhou Province (Hapuarachchi et al. 2019; Luangharn et al. 2021; He et al. 2022; Sun et al. 2022). These studies show that there is an unrecognized diversity of *Ganoderma* species in southwest China. More potential new species of *Ganoderma* may be discovered in the future.

In this study, two new species viz *G.phyllanthicola* and *G.suae* from Southwest China are introduced based on morphology and multigene phylogeny. *Ganodermaphyllanthicola* and *G.suae* satisfied the generic concept of the genus *Ganoderma* (Karsten 1881). They comprise subglobose to ellipsoid or ovoid basidiospores, truncated, double-walled with thick walls, exospore wall semi-reticulate, endospore wall smooth or with conspicuous spinules, homogeneous or heterogeneous context and laccate with variable ornamentation pileus surface. When compared with each other, *G.phyllanthicola* and *G.suae* occupied distinct and distant positions in the multilocus phylogenetic tree, and the morphology of their basidiomata also exhibits distinct macro- and microscopic characters that can further differentiate the two species. Thus, based on convergent results from morphology and molecular data analyses, *G.phyllanthicola* and *G.suae* are considered to be new species to science.

*Ganodermaphyllanthicola* was closely related to *G.castaneum*, *G.philippii* and G. *tropicum* in the phylogeny inferred from the concatenated sequence data set. Morphologically, they are easily distinguishable by some macro- and microscopic characters of their basidiomata. Contrary to *G.phyllanthicola*, *G.castaneum* has a broadly attached, flabelliform, chestnut brown pileus surface with wide concentric ridges, heterogeneous context, regular palisade pileipellis cells, and broadly ellipsoid basidiospores not obviously truncated with smooth endospore walls (Sun et al. 2022; Table 2). *Ganodermaphilippii* and *G.tropicum*, contrary to the new species, is characterized by flabelliform to circular, non-coriaceous basidiomata and very much smaller basidiospores (Steyaert 1972; Moncalvo and Ryvarden 1997; Table 2). Moreover, *Ganodermaenigmaticum* can be easily distinguished from *G.phyllanthicola* by the stipitate basidiomata and regular pileipellis cells (Coetzee et al. 2015). *Ganodermaorbiforme* has biannual or perennial basidiospores and longer pileipellis cells than those of *G.phyllanthicola* (Ryvarden 2000; Table 2).

**Table 2.** Morphological comparison of *Ganodermaphyllanthicola* sp. nov., and *G.suae* sp. nov., with their closest relatives in the combined phylogeny.

Species	Shape	Context	Pileipellis cells	Pores (per mm)	Basidiospores (μm)	Reference
*Ganodermaaridicola*	Sessile, dimidiate	2.4–3 µm thick, homogeneous, fuscous	cells clavate, 30–55 × 5–8 μm	6–8	9.7–11.2 × 7.0–7.8	Xing et al. 2016
*G.castaneum*	sessile, flabelliform	up to 1.6 cm thick, heterogeneous, the upper layer pale straw yellow, the lower layer dark brown,	composed of regular palisade, clavate end cells 25–40 × 3–5 μm	4–6	6.2–8.5 × 4.2–6.3	Sun et al. 2022
***G.phyllanthicola***	**sessile, sub-circular, flabelliform to shell-shaped**	**up to 0.8 cm thick, homogeneous, cinnamon brown to chestnut brown**	**composed of irregular, narrowly clavate end cells, straight to flexuous or irregular, 15–33 × 5–9 μ**m	**5–7**	**8.5–11.0 × 6.0–7.5**	**this study**
*G.enigmaticum*	stipitate, globular	context soft, homogenous, dark brown	amyloid elements 20–46 × 5.5–9 um	3–5	8.0–11.0 × 3.5–6.0	Coetzee et al. 2015
*G.lucidum*	stipitate to sessile	thinner context of white to slightly cream color context	amyloid hyphal end cells up to 7–11 μm diam	4–5	7.7–11.5 × 5.2–8.4	Ryvarden and Gilbertson 1993
*G.multiplicatum*	sessile, flabelliform, applanate or convex	up to 2 cm thick, homogeneous, cinnamon colour, darker toward the tubes	cells clavate, cylindrical or irregular, 38–65 × 5.6–10 µm	5–6	6.0–10.0 × 4.5–7.0	Gottlieb and Wright 1999
*G.orbiforme*	sessile, flabelliform or spathulate	context up to 0.4–1.0 cm thick, triplex	composed of apically acanthus like branched cells, 50–100 X 6–12 μm	4–7	7.1–12.6 × 5.2–7.7	Ryvarden 2000; Wang et al. 2014
*G.philippii*	sessile, flabelliform to circular	up to 1.4 cm thick, homogeneous, brown	–	5–6	6.0–8.0 × 3.0–4.0	Steyaert 1972, Moncalvo and Ryvarden 1997
*G.polychromum*	sessile to o substipitate, flabelliform	pink buff to cinnamon buff concentric growth zones	–	4–5	10.3–18.3 × 7.0–11.9	Murrill 1908
*G.resinaceum*	sessile to stipitate, round-flabelliform	0.4-1.3 cm thick, homogeneous context, wood-coloured to pale tawny brown., with resinous incrustations	cells clavate, narrowly clavate, or almost cylindrical, 34–59 × 6.2–9.3 μm,	3–4	9.0–13.0 × 6.0–8.0	Steyaert 1972; Ryvarden 2000; Náplavová et al. 2020
*G.sessile*	sessile, pileus sometimes imbricate, conchate to flabelliform	context thin, soft corky or woody, radially fibrous, concentrically zonate, ochraceous	cylindric, smooth elements, 60–75 × 7–10 µm	4–5	12.0–16.0 × 6.0–8.0	Murrill 1902; Gottlieb and Wright 1999
***G.suae***	**sessile to substipitate, variable, reniform**	**up to 0.8 cm thick, heterogeneous, the upper layer greyish white, the lower layer cinnamon brown, without resinous incrustations**	**cells clavate, 24–43 × 6–11 μm**	**4–6**	**8.0–10.5 × 5.0–7.0**	**this stud**y
*G.tropicum*	usually sessile, sometimes laterally stipitate, flabelliform to shell-shaped or circular	up to 2.2 cm thick, homogeneous, dark brown	cells clavate, sometimes branched or protuberant, inflated and flexuous, 19–32 × 4–9 μm	4–6	6.8–10.0 × 4.0–6.4	Ryvarden 1981
*G.vivianimercedianum*	sessile to substipitate, flabelliform in pole view	1–1.5 cm thick, homogeneous, caramel above and dark brown toward the tubes,	cells clavate, apex occasionally slightly widened, 36–65 × 7.2–12 µm	3–5	9.0–12.0 × 6.0–8.0	Torres-Torres et al. 2008
*G.zonatum*	sessile, applanate to convex	homogeneous, slightly zonate, dark brow	cells cylindrical to clavate, 30–70 × 5–12 µm	4–5	12.0–14.0 × 6.0–9.0	Loyd et al. 2018

*Ganodermaresinaceum* is known to be a Northern Hemisphere species, mainly occurring in Europe (Patouillard 1889; Moncalvo et al. 1995; Ryvarden and Melo 2014). The European specimens are easily recognized in the field by thick, soft and pale context. The first signs of genetic diversity within *G.resinaceum* were observed by Moncalvo (2000), and Loyd et al. (2018) showed that G.resinaceum sensu American auctores encompassed at least two distinct species, viz. *G.polychromum* and *G.sessile*. Cabarroi-Hernández et al. (2019) studies confirmed that *G.resinaceum* sensu auctores from China, East Africa, Europa, and both North and South America represented a species complex. Study of phylogenetic inferences based on multilocus sequences by Hernand éz et al. (2019) also showed that *G.resinaceum* represents a species complex.

Our results based on polygenic phylogenetic analysis also confirm that *Ganodermaresinaceum* represents a species complex, encompassing several distinct species, namely *G.platense*, *G.polychromum*, *G.sessile*, and *G.suae*. *Ganodermasuae* emerges as a newly recognized species within the *G.resinaceum* sensu complex group (Fig. 1). *Ganodermasuae* is characterized by its annual basidiomata, reddish brown to oxblood red pileus surface, heterogeneous context without resinous incrustations (without black melanoid lines), wavy margin and almond-shaped basidiospores not obviously truncated, endospore walls with dense spinules (8.0–10.5 × 5.0–7.0 μm), can be easily distinguished from *G.resinaceum* (Ryvarden 2000). Náplavová et al. 2020 confirmed the presence of two distinct genotypes (genotype A and genotype B) in European *G.resinaceum* by comparing partial sequences of the TEF1-α region and the 25 s LSU rRNA gene. Their study also showed that basidiospore sizes range between 9.6–14.4 × 6.0–8.4 µ m in genotype A and 6–12.0 × 7.2–9.6 µ m in genotype B. Besides, specimens of both genotypes share the same pileus surface (glossy with resinous layer) and almost identical coloration. Only the context color was lighter brown beige to sand yellow in genotype A and darker brown beige to ochre brown in genotype B. *Ganodermaresinaceum* from Europe has a special laccate and glossy with resinous layer pileus surface, homogeneous context, and larger basidiospores cells than those of *G.suae*. (Ryvarden 2004; Náplavová et al. 2020). Thus, *Ganodermasuae* from China and *G.resinaceum* from Europe should be recognized as two different species. Table 2 presents a morphological comparison between the new species and its closest phylogenetic neighbors. Although we are of the opinion that *G.suae* well represent a species on its own, more material, ideally from various localities, and DNA sequences, is necessary to reveal the species diversity and kinship of *G.resinaceum* complex groups.

Recent studies have shown that the specimen *G.resinaceum* collected from China is inconsistent with the original description; therefore, it is clear that *G.resinaceum* is not distributed in China (Sun et al. 2022). It’s noteworthy that we have also collected a sample (HL199) from Yunnan Province, which differs from both *G.resinaceum* and *G.suae* in terms of its distinct macro-morphology and multi-gene sequences. Regrettably, the specimens were sterile and micromorphological data were missing. In the future, collecting additional specimens will be crucial for revealing the true distribution and diversity of *G.resinaceum* complex groups in China.

## Supplementary Material

XML Treatment for Ganoderma phyllanthicola

XML Treatment for Ganoderma suae

## ﻿Additional information

### Author ORCIDs

Jun He https://orcid.org/0000-0001-7027-7206

Wan-Zhong Tan https://orcid.org/0000-0002-9355-5798

Zong-Long Luo https://orcid.org/0000-0001-7307-4885

### Data availability

All of the data that support the findings of this study are available in the main text or Supplementary Information.

## Supplementary materials

Supplementary material 1 Phylogenetic tree Data type pdf Explanation note Maximum likelihood (ML) tree is based on combined ITS + nrLSU + TEF1-α + RPB2 sequence data. Bootstrap support values with a maximum likelihood (ML) greater than 60% and Bayesian posterior probabilities (PP) greater than 0.90 given above the nodes, shown as “ML/PP”. https://binary.pensoft.net/file/1074149This dataset is made available under the Open Database License (http://opendatacommons.org/licenses/odbl/1.0/). The Open Database License (ODbL) is a license agreement intended to allow users to freely share, modify, and use this Dataset while maintaining this same freedom for others, provided that the original source and author(s) are credited.Author: Jun He
