# Supplementary figures and images for: ﻿Two new species of Ganoderma (Ganodermataceae, Basidiomycota) from Southwest China
Source: MycoKeys. 2024 Jun 19;106:97–116. doi: 10.3897/mycokeys.106.121526 (PMC11208776; doi:10.3897/mycokeys.106.121526)

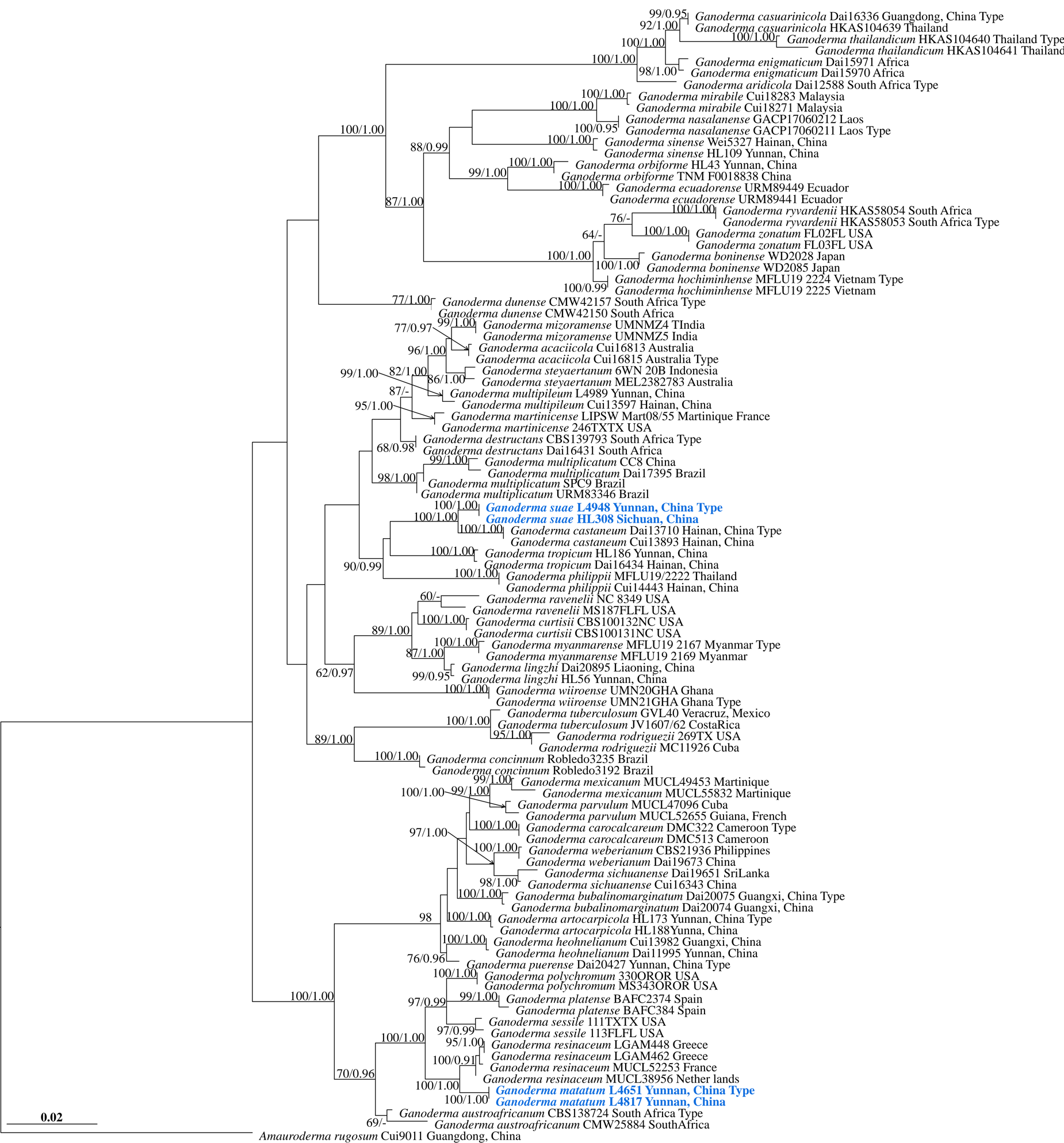

Supplement: Supplementary material 1 — Phylogenetic tree [file mycokeys-106-097-s001.pdf]
